# Gastric Mixed Neuroendocrine Non-Neuroendocrine Neoplasms: A Western Center Case Series

**DOI:** 10.3390/medsci9030047

**Published:** 2021-06-25

**Authors:** Marcus Fernando Kodama Pertille Ramos, Marina Alessandra Pereira, Arthur Youssif Mota Arabi, Melissa Mello Mazepa, Andre Roncon Dias, Ulysses Ribeiro, Bruno Zilberstein, Sergio Carlos Nahas

**Affiliations:** Faculdade de Medicina, Instituto do Cancer, Hospital das Clinicas HCFMUSP, Universidade de Sao Paulo, Sao Paulo 01246-000, Brazil; marina.pereira@hc.fm.usp.br (M.A.P.); arthur.arabi@hc.fm.usp.br (A.Y.M.A.); melissa.mazepa@hc.fm.usp.br (M.M.M.); andre.dias@hc.fm.usp.br (A.R.D.); ulysses.ribeiro@hc.fm.usp.br (U.R.J.); bruno.zilberstein@hc.fm.usp.br (B.Z.); sergio.nahas@hc.fm.usp.br (S.C.N.)

**Keywords:** stomach neoplasms, mixed non-neuroendocrine neuroendocrine neoplasms, MiNENs, mixed adeno-neuroendocrine carcinoma, MANEC

## Abstract

Background: Mixed neuroendocrine non-neuroendocrine neoplasms (MiNENs) represent a rare tumor composed of adenocarcinoma and neuroendocrine carcinoma components. This study reports a case series of gastric MiNEN and discusses issues related to its diagnosis, management, and outcomes. Methods: We retrospectively analyzed data from patients with gastric MiNEN who underwent surgical resection at our service from 2009 to 2020. Patients with gastric adenocarcinoma served as a comparison group. Clinical, pathologic, and surgical characteristics were compared. Results: During the selected period, 5 gastric MiNEN patients and 597 patients with gastric adenocarcinoma were included. Among the clinical variables, age, sex, BMI, and laboratory exams were similar between the two groups. Only ASA classification was different (*p* = 0.015). Pathological variables such as tumor size, lymphovascular invasion, number of retrieved lymph nodes, and pTNM staging were also similar between both groups. Lastly, early surgical outcomes and long-term survival did not differ between gastric MiNEN and adenocarcinoma patients. Conclusion: A MiNEN is a rare tumor that represents less than 1% of GC patients undergoing curative treatment, and demonstrated clinicopathological characteristics and outcomes similar to gastric adenocarcinoma.

## 1. Introduction

The mixed neuroendocrine non-neuroendocrine neoplasms (MiNENs) are rare neoplasms that are defined as mixed epithelial neoplasms composed of both neuroendocrine and non-neuroendocrine components. The new term “MiNEN” was proposed in 2019 to replace the old terminology used by the World Health Organization (WHO) Classification of Tumors of the Digestive System for the stomach and any site within the gastro-entero-pancreatic tract, previously named as “mixed adeno-neuroendocrine carcinomas” (MANECs) [1,2,3].

At least 30% of either component should be identified to qualify for this definition. This threshold is based mainly on the assumption that a lesser represented component (less than 30%) is unlikely to influence the biological behavior of the whole neoplasm. Therefore, the precise diagnosis depends on histopathological and immunohistochemical examination [4,5,6].

MiNEN has been identified in various organs, including the pancreas, colon, biliary tract, and stomach. Due to its rarity, the prognosis of patients with MiNEN remains controversial, and the literature shows conflicting results [4,7]. In general, they have been associated with aggressive behavior, strong invasiveness, and high lymph node dissemination [8]. It was suggested that MiNEN patients may have a worse prognosis when compared to those with isolated gastric adenocarcinoma and neuroendocrine carcinoma [2].

In addition, due to its low incidence, some debates regarding which would be the best therapeutic option still exist [2,6,8,9]. Based on the WHO recommendations, it is suggested that MiNEN should be treated as adenocarcinoma [10]. However, some authors reported that treatment should be based on the most aggressive histologic component [6].

Thus, the present study aims to describe a case series of patients with gastric MiNENs treated in a reference western center and discuss the main issues related to this particular type of tumor.

## 2. Materials and Methods

### 2.1. Patients

All gastric cancer patients who underwent gastrectomy with curative intent between 2009 and 2020 were retrospectively reviewed. All data were obtained retrospectively from a database maintained at our institution. Patients with histopathological diagnosis of MiNEN were included. As a comparison group, patients with gastric adenocarcinoma in the same period were selected. Patients with pathological diagnoses of other gastric tumors such as GISTs, lymphoma, and isolated neuroendocrine tumors were excluded.

**Figure 1:**
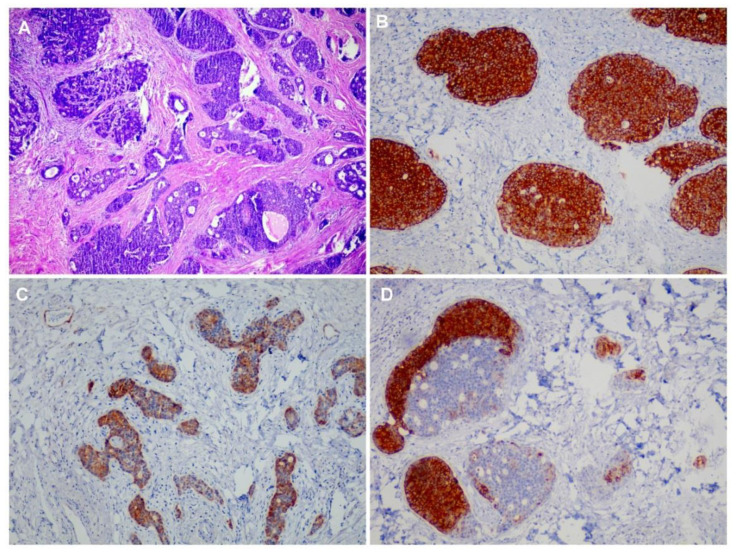
Microscopic findings of MiNEN in the stomach with the neuroendocrine component and epithelial part of the tumor: (**A**) H&E stain; (**B**) tumor cells marked by neuroendocrine marker synaptophysin; (**C**) immunohistochemistry for CK7; and (**D**) CK20 showing positivity for epithelial component—(20× magnification).

The pathologic diagnosis of MiNEN was established by the presence of at least 30% of each component (both adenocarcinoma and neuroendocrine carcinoma), according to the World Health Organization (WHO) classification [5,7]. Immunostaining with specific neuroendocrine markers (chromogranin A, synaptophysin, CD56, CK35BH11) and markers of non-endocrine differentiation (adenocarcinoma component) (AE1/AE3, CDX2, CK7, CK20) was performed to confirm the presence of both histological patterns. Figure 1 shows some histopathological patterns found in some patients of the MiNEN group.

The evaluated data included sex, age, body mass index (BMI), serum levels of hemoglobin and albumin, neutrophil-lymphocyte ratio (NLR) [11], the presence of comorbidities according to the Charlson–Deyo comorbidity index (CCI) [12], and the physical status classification of the American Anesthesiology Society (ASA) [13].

The preoperative staging was performed by using computed tomography (CT) scan, laboratory exams, and upper gastrointestinal endoscopy. All patients were staged according to the 8th edition of the American Joint Committee on Cancer (AJCC) staging system [5,14]. Postoperative complications (POC) were assessed using the Clavien–Dindo grading system, and POC grade ≥ 3 was classified as a major complication [15].

All surgeries were carried out by experienced surgeons, following the guidelines of the Japanese Gastric Cancer Association and the recommendations of the Brazilian Gastric Cancer Association Consensus [16,17].

### 2.2. Statistical Analysis

The descriptive statistics included frequency with percentage for nominal variables and mean (with standard deviation) for the continuous variables. Fisher’s exact test was used for categorical data and the T-test was used for continuous data. The disease-free survival (DFS) and the overall survival (OS) were estimated using the Kaplan–Meier method, and the differences between survivals were examined by the Log Rank Test. The survival time was calculated from the date of the surgery to the date of death or recurrence. The living patients were censored on the date of the last follow-up. All *p*-values < 0.05 were considered statistically significant. The analysis was performed by the software SPSS, version 20.0 (SPSS Inc., Chicago, IL, USA).

## 3. Results

During the referred period, 602 patients who underwent gastrectomy were included in the study. Of these, five patients (0.8%) were diagnosed with gastric MiNEN. The remaining 597 patients (99.2%) comprised patients diagnosed with gastric adenocarcinoma.

The clinical and surgical characteristics of both groups are described in Table 1. The mean age of the patients in the MiNEN group was 65.8 years (range from 50 to 83), with a male predominance. Among the clinical variables evaluated, ASA III/IV was more frequent in the MiNEN group (*p* = 0.015). There was no significant difference between both groups for the other clinical variables evaluated.

**Figure 2:**
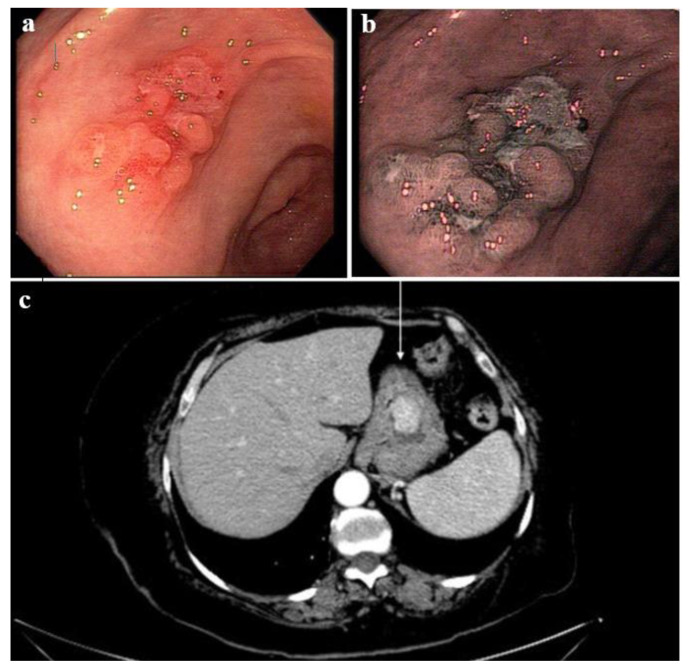
(**a**,**b**) Upper digestive endoscopy demonstrating gastric lesions compatible with MiNEN in one patient (**a**) the hyperemic surface of the MiNEN, associated with a central depression; (**b**) chromoendoscopy with magnification showing visible subepithelial capillaries; (**c**) CT scan showing gastric MiNEN in the gastric body of a patient.

According to the location of MiNEN, two were located at the antrum, two at the body, and one in the gastric fundus. Figure 2 demonstrates tomographic and endoscopic features encountered in some of the MiNEN patients.

Regarding surgical treatment, one patient in the MiNEN group had a laparoscopic intervention and four underwent open gastrectomy. All surgical procedures had free margins. The type of gastrectomy was subtotal in most cases (four patients).

Table 2 shows the pathologic characteristics of MiNEN compared with gastric adenocarcinoma. The groups were similar concerning the pT, pN, and pTNM stage. The mean number of lymph nodes retrieved in the MiNEN group was 32.8, and three patients had lymph node metastasis identified on the histopathological analysis. Most cases in MiNEN were pT3/pT4 (60%).

The immunohistochemistry analysis was performed in the MiNEN group to confirm the presence of both neuroendocrine and adenocarcinoma cells markers. Chromogranin A, synaptophysin, CD56, CK35BH11 were used for the neuroendocrine lineage and AE1/AE2, CDX2, CK7, CK20 to confirm the existence of adenocarcinoma cells. The characteristics of the five MiNEN patients, including histological and immunohistochemical findings, are summarized in Table 3.

Regarding postoperative results (Table 4), the mean length of hospital stay in MiNEN patients was 16.3 days (range from 6 to 38). Mortality at 30 and 90 days in the MiNEN group was 0% and 20%, respectively, with no statistical difference compared to the adenocarcinoma group. Postoperative chemotherapy was performed on only one patient with capecitabine and oxaliplatin. The median follow-up for the entire cohort was 36 months (range from 1 to 119 months). Among patients diagnosed with MiNEN, one had disease recurrence (located in the liver) and two died. There was also no significant difference concerning survival between groups.

## 4. Discussions

Mixed neuroendocrine non-neuroendocrine neoplasms (MiNENs) represent a rare diagnosis that has been described in several organs, including the stomach, and are histologically characterized by epithelial neoplasms that display a coexistence of neuroendocrine and non-neuroendocrine histology [2].

Historically, MiNENs are considered rare and aggressive tumors [18], and the neuroendocrine component behavior seems to be the main factor that defines their clinical evolution [2,8,9]. In addition, the heterogeneous behavior of this type of tumor and its difficult diagnosis, associated with the rapid growth, high risk of lymph node metastasis, and strong invasion capability, contribute to the poor prognosis of the MiNEN patients, when compared to those who have only adenocarcinoma cells [2,8,19].

In our study, we described a case series involving patients diagnosed with MiNEN that underwent surgical resection, and we compared it to a cohort of patients with gastric adenocarcinoma. We found a frequency of 0.8% of MiNEN in GC who underwent surgical resection. This result is difficult to compare with the current literature due to its rarity [1,2]. Additionally, according to our analysis, the initial clinicopathological characteristics of both groups were similar and they had similar outcomes with no differences in survival.

The physiopathology of MiNEN is still a point that remains uncertain, such as its genetic characteristics. This fact negatively impacts the pursuit of establishing innovative and effective therapeutic guidelines [7,20]. Two theories discuss the MiNEN genetic origin: some argue that both tumor cell lineages have different origins, with the adenocarcinoma cells being differentiated from multipotent stem-cells, while the neuroendocrine cells have their origin in embryonic neural cells. Nonetheless, other authors claim that both tumor cells have a common origin, being differentiated from a multipotent stem-cell clonal lineage [2,3,21]. This last theory seems to be the most accepted nowadays, after recent discoveries of an overlapping mutational profile in both cell varieties that compose the MiNEN [20].

The most important factor related to the diagnosis of MiNEN is histological analysis [19]. To determine the histopathological diagnosis of MiNEN, it is necessary to find the presence of both adenocarcinoma and neuroendocrine tumor cells [8,21]. Additionally, complementary exams with immunohistochemical staining are necessary to support the histological diagnosis. Many cases of MiNENs are still misdiagnosed, and a great part of this is explained by the poor choice of parameters of the histopathological evaluation. Indeed, this misdiagnosis could be overcome by routinely using immunohistochemistry analysis, searching specific markers of both neuroendocrine and non-neuroendocrine tumor cells [6,8,22]. In our study, an immunohistochemistry panel with specific markers was performed in all MiNEN cases, evidencing the presence of both neuroendocrine components (chromogranin A, synaptophysin, CD56, CK35BH11) and adenocarcinoma cells (AE1/AE2, CDX2, CK7, CK20).

The complete diagnostic investigation of a patient with MiNEN involves performing an upper gastrointestinal endoscopy and a contrast-enhanced CT scan [19]. In CT analysis, the neuroendocrine carcinoma images tend to appear as isodense lesions at the pre-contrast phase, with marked enhancement after the administration of intravenous contrast in the arterial phase, as demonstrated in Figure 2 in this study [23,24,25]. With regard to the endoscopic aspect, MiNENs often present with typical appearances: in white-imaging endoscopy, it is usually possible to see the mucosal hyperemia in the lesion area, commonly associated with a central depression; when using endoscopic magnification, a central discoloration linked to visible subepithelial capillaries is typically noted, making a corkscrew pattern [26]. Therefore, NECs are usually seen on endoscopy imaging as a solitary lesion, generally with at least 2 cm in its greater diameter, and most commonly located in the gastric body or fundus [27].

Due to MiNEN’s characteristic of being rare and having heterogeneous behavior, there are still some disagreements regarding the best therapeutic approach [8]. As gastric adenocarcinoma, the surgical resection associated with lymphadenectomy is the standard treatment for MiNEN. Some also showed that adjuvant chemotherapy seems to have an important role in the therapeutic arsenal [8,27]. Conversely, others reported that there was no survival benefit in patients who received adjuvant chemotherapy with platinum-based regimens for gastric MiNENs [4]. Indeed, in the absence of data from clinical trials, MiNENs are commonly treated according to the standard of care for pure neuroendocrine carcinomas or gastric adenocarcinomas.

Unfortunately, the diagnosis of MiNEN is usually late. Generally, MiNENs show nonspecific gastric cancer symptoms and are often diagnosed in advanced disease stages, including the presence of distant metastasis at the time of diagnosis, which justifies the fact that some authors claim that MiNEN tends to have a considerably poor prognosis [28,29]. On the other hand, some articles argue that these patients have a similar prognosis to those with gastric adenocarcinoma [27]. As previously discussed, in our study, there were no significant differences related to overall survival between MiNEN and adenocarcinoma patients.

This study has some limitations. First, we included in both groups only patients with localized oncologic disease who received surgical resection. Thus, palliative cases with metastatic disease were not included, adding a selection bias to the study. Additionally, due to its rare occurrence, the number of patients with gastric MiNENs in our cohort is low, which limits some statistical analyses and, therefore, the establishment of more detailed conclusions about the survival and prognosis in these cases.

Despite all these obstacles, this cohort is representative of real-world clinical practice. We conducted the presented study in a reference center in the treatment of oncologic patients in our country [30], containing experienced professionals in managing those cases. In addition, our study sought to discuss current aspects of this rare disease to bring the most current evidence of its diagnosis and management.

## 5. Conclusions

MiNEN is a rare tumor that represents less than 1% of GC patients who undergo curative resection. Patients with MiNEN had clinicopathological characteristics and outcomes similar to those with gastric adenocarcinoma. Due to their dual histological profile, IHC for neuroendocrine and epithelial markers is essential for diagnostic confirmation.

## Figures and Tables

**Table 1:** Clinical and surgical characteristics of patients with gastric adenocarcinoma and MiNEN.

Variables		Gastric Adenocarcinoma	MiNEN	
		*n* = 597 (%)	*n* = 5 (%)	*p*
**Sex**				0.334
	Female	247 (41.4)	1 (20)	
	Male	350 (58.6)	4 (80)	
**Age (years)**				0.589
	Mean (SD)	62.8 (12.6)	65.8 (11.7)	
**Body mass index (Kg/m²)**				0.053
	Mean (SD)	24.6 (5.0)	29.6 (7.9)	
**Hemoglobin (g/dL)**				0.791
	Mean (SD)	12.5 (5.5)	11.8 (3.5)	
**Albumin (g/dL)**				0.573
	Mean (SD)	4.0 (1.4)	4.4 (0.3)	
**Neutrophil-lymphocyte ratio (NLR)**				0.778
	Mean (SD)	2.74 (2.64)	3.07 (1.05)	
**Charlson–Deyo Comorbidity Index (CCI)**				0.054
	0	389 (65.2)	1 (20)	
	≥1	208 (34.8)	4 (80)	
**ASA (American Society of Anesthesiologists)**				0.015
	I/II	450 (75.4)	1 (20)	
	III/IV	147 (24.6)	4 (80)	
**Type of Gastrectomy**				0.662
	Subtotal	387 (64.8)	4 (80)	
	Total	210 (35.2)	1 (20)	
**Surgical access**				1.0
	Open	491 (82.2)	4 (80)	
	Laparoscopic	106 (17.8)	1 (20)	
**Lymphadenectomy**				0.204
	D1	101 (16.9)	2 (40)	
	D2	496 (83.1)	3 (60)	

SD: standard deviation. *p* values in bold are statistically significant

**Table 2:** Pathological characteristics of patients with gastric adenocarcinoma and MiNEN.

Variables		Gastric Adenocarcinoma	MiNEN	
		*n* = 597 (%)	*n* = 5 (%)	*p*
**Tumor size (cm)**				0.879
	Mean (SD)	4.7 (3.0)	4.5 (3.4)	
**Lymphatic Invasion**				1.0
	Absent	308 (51.6)	3 (60)	
	Present	289 (48.4)	2 (40)	
**Venous Invasion**				1.0
	Absent	402 (67.3)	3 (60)	
	Present	195 (32.7)	2 (40)	
**Perineural invasion**				0.378
	Absent	317 (53.1)	4 (80)	
	Present	280 (46.9)	1 (20)	
**pT status**				1.0
	pT1/T2	251 (42)	2 (40)	
	pT3/T4	346 (58)	3 (60)	
**Number of Lymph node dissected**				0.329
	Mean (SD)	40.7 (18.1)	32.8 (17.7)	
**pN status**				1.0
	pN0	262 (43.9)	2 (40)	
	pN+	335 (56.1)	3 (60)	
**pTNM**				0.395
	I/II	337 (56.4)	4 (80)	
	III/IV	260 (43.6)	1 (20)	

SD: standard deviation.

**Table 3:** Clinicopathological characteristics, surgical and oncological outcomes of the five MiNEN patients included in the study.

	Number of Case				
Variables	1	2	3	4	5
**Sex**	Female	Male	Male	Male	Male
**Age (years)**	69.6	82.7	50.8	61.1	65.0
**Tumor Location**	middle third	middle third	lower third	middle third	lower third
**Tumor size (cm)**	3.0	1.8	3.2	10.6	4.0
**Neuroendocrine vs. Adenocarcinoma (%)**	70% vs. 30%	70% vs. 30%	60% vs. 40%	70% vs. 30%	70% vs. 30%
**IHC(+) for Adenocarcinoma component**	CK7	CK7, CK20, and CDX-2	CK7 and CK20	AE1/AE3	AE1/AE3
**IHC(+) for Neuroendocrine component**	Chromogranin A and Synaptophysin	Chromogranin A, Synaptophysin, and CK35BH11	Chromogranin A and Synaptophysin	Chromogranin A, Synaptophysin, and CK35BH11	Chromogranin A and Synaptophysin
**Mitotic count per 10 HPFs**	—	5	1	—	20
**Ki67 labeling index (%)**	15	5	80	40	70
**Presence of necrosis**	Absent	Absent	Absent	Present	Absent
**Lymphatic Invasion**	Present	Present	Present	Absent	Absent
**Venous Invasion**	Present	Present	Present	Absent	Absent
**Perineural invasion**	Absent	Present	Absent	Present	Absent
**LN+/LNs total**	0+/46	1+/5	2+/26	2+/40	0+/47
**pTNM**	T4a N0 M0	T4a N1 M0	T1b N1 M0	T3 N1 M0	T1b N0 M0
**Final Stage**	IIB	IIIA	IB	IIB	IA
**DFS (months)**	1.3	8.7	37.0	54.2	17.3
**OS (months)**	1.3	16.7	37.0	54.2	17.3
**Patient status**	Death	Death	Alive, without disease	Alive, without disease	Alive, without disease

IHC, immunohistochemistry; HPFs, high-power fields; LN, lymph node; DFS, disease-free survival; OS, overall survival.

**Table 4:** Surgical outcomes and follow-up of patients with gastric adenocarcinoma and MiNEN.

Variables		Gastric Adenocarcinoma	MiNEN	
		*n* = 597 (%)	*n* = 5 (%)	*p*
**Length of hospital stay (days)**				0.371
	Mean (SD)	12.0 (9.4)	16.3 (14.7)	
**Postoperative complication (POC)**				0.022
	No POC/Minor POC	515 (86.3)	2 (40)	
	Major POC	82 (13.7)	3 (60)	
**30-day mortality**				1.0
	No	576 (96.5)	5 (100)	
	Yes	21 (3.5)	0 (0)	
**90-day mortality**				0.311
	No	555 (93)	4 (80)	
	Yes	42 (7)	1 (20)	
**Chemotherapy**				0.204
	No	288 (48.2)	4 (80)	
	Yes	309 (51.8)	1 (20)	
**Recurrence**				—
	No	468 (78.4)	4 (80)	
	Yes	129 (21.6)	1 (20)	
**Death**				—
	No	390 (65.3)	3 (60)	
	Yes	207 (34.7)	2 (40)	
**Disease-free survival rate**				0.925 *
	DFS (%)	71.3	71.7	
**Overall survival rate**				0.612 *
	OS (%)	56.7	60.0	

SD: standard deviation, *p* values in bold are statistically significant, * p Log Rank.

## Data Availability

The dataset analyzed for this study is available from the corresponding author upon reasonable request.
